# Otx2 ChIP-seq Reveals Unique and Redundant Functions in the Mature Mouse Retina

**DOI:** 10.1371/journal.pone.0089110

**Published:** 2014-02-18

**Authors:** Alexander Samuel, Michael Housset, Bruno Fant, Thomas Lamonerie

**Affiliations:** Institut de Biologie Valrose, University of Nice Sophia Antipolis, CNRS UMR7277, Inserm U1091, Nice, France; University of Cologne, Germany

## Abstract

During mouse retinal development and into adulthood, the transcription factor Otx2 is expressed in pigment epithelium, photoreceptors and bipolar cells. In the mature retina, *Otx2* ablation causes photoreceptor degeneration through a non-cell-autonomous mechanism involving *Otx2* function in the supporting RPE. Surprisingly, photoreceptor survival does not require *Otx2* expression in the neural retina, where the related *Crx* homeobox gene, a major regulator of photoreceptor development, is also expressed. To get a deeper view of mouse *Otx2* activities in the neural retina, we performed chromatin-immunoprecipitation followed by massively parallel sequencing (ChIP-seq) on Otx2. Using two independent ChIP-seq assays, we identified consistent sets of Otx2-bound cis-regulatory elements. Comparison with our previous RPE-specific Otx2 ChIP-seq data shows that Otx2 occupies different functional domains of the genome in RPE cells and in neural retina cells and regulates mostly different sets of genes. To assess the potential redundancy of Otx2 and Crx, we compared our data with Crx ChIP-seq data. While Crx genome occupancy markedly differs from Otx2 genome occupancy in the RPE, it largely overlaps that of Otx2 in the neural retina. Thus, in accordance with its essential role in the RPE and its non-essential role in the neural retina, Otx2 regulates different gene sets in the RPE and the neural retina, and shares an important part of its repertoire with Crx in the neural retina. Overall, this study provides a better understanding of gene-regulatory networks controlling photoreceptor homeostasis and disease.

## Introduction

A handful of signalling pathways and transcription factors families are reused for multiple purposes in plant and animal development [1], implying that these actors take part in different regulatory networks. Transcription factors act in this context by regulating target genes through interaction with protein partners and binding to selected cis-regulatory sequences. Although significant progress has been made in the large-scale identification of cis-regulatory elements[2], we are still lacking a global view of how a defined transcription factor regulates various target genes in different contexts.

All three members of the Otx family of homeodomain transcription factors, Otx1, Otx2 and Crx play critical roles in development and function of the mammalian retina. Mutations in human *OTX2* and *CRX* are associated with severe ocular and retinal diseases such as microphthalmia, retinitis pigmentosa, cone-rod distrophy and Leber’s congenital amaurosis [3]–[5]. Early in mouse retinal development at embryonic day 9.5 (E9.5), overlapping expression of *Otx1* and *Otx2* is required to specify the retinal pigment epithelium (RPE) [6]. Otx2 expression is then maintained in RPE cells into adulthood. At E12.5, photoreceptor and bipolar cell fate is determined by the expression of Otx2 in retinal progenitor cells [7], which controls the subsequent induction of the related Crx transcription factor. Expression of both Otx2 and Crx is then maintained throughout life in photoreceptor and bipolar cells [8]–[10].

The function of *Otx2* has been addressed in the adult eye. At this stage, RPE cells express *Otx2* alone while photoreceptor and bipolar cells co-express *Otx2* and *Crx*. Photoreceptor cells express *Crx* at a higher level than *Otx2* while bipolar cells, express *Otx2* at a higher level than *Crx* [11]. Conditional ablation in all cell types expressing *Otx2* causes exclusive, progressive and complete degeneration of photoreceptors [9]. Strikingly, a similar phenotype is observed when *Otx2* deletion is restricted to RPE cells, indicating a non-cell-autonomous, RPE-based mechanism for photoreceptor degeneration. Furthermore, RPE-specific expression of *Otx2* in conditionally ablated retinas rescues photoreceptor disease, showing that *Otx2* expression in photoreceptor and bipolar cells is not required cell-autonomously for survival [12]. Interestingly, gene expression analysis following *Otx2* knockout in the whole retina has identified mostly RPE-specific genes as *Otx2* direct target genes. Despite the recognized role of *Otx2* in photoreceptor cell specification and development and the maintenance of its expression in photoreceptor and bipolar cells, the function of *Otx2* in the adult neural retina remains unknown.

Here, genome-scale identification of Otx2 binding sites in the neural retina and comparison with Otx2 binding sites in the RPE was done as a first step toward the characterization of Otx2 target genes in both compartments. Otx2 genome occupancy was analysed using two independent Otx2 chromatin immuno-precipitation strategies followed by massively parallel sequencing (ChIP-seq), providing a robust map of Otx2 binding sites in both compartments of the adult retina. We show that cis-regulatory regions bound by Otx2 in the RPE and in the neural retina are mostly non-overlapping and have very different structural features, indicating tissue specific activities. Finally, comparison to Crx genome occupancy clearly points to redundant Otx2 and Crx functions in the neural retina.

## Results

### Distinct patterns of Otx2 genome occupancy in retinal compartments

**Figure 1:**
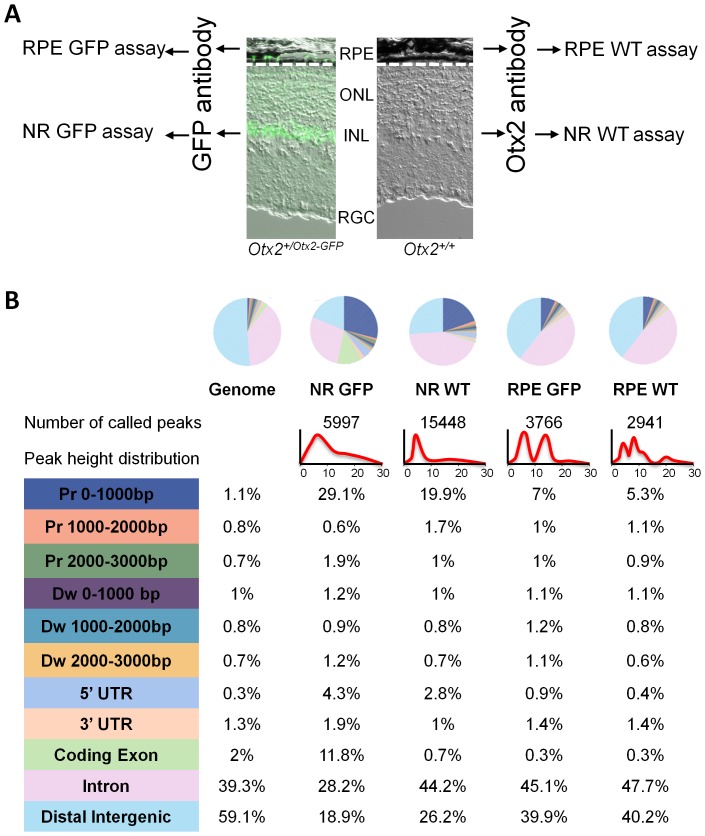
Tissue-specific Otx2 genome binding in the retina. **A.** Experimental design: four independent ChIP experiments were performed. RPE and neural retine (NR) nuclei of *Otx2^+/Otx2−GFP^* mice were subjected to the GFP assays using a GFP antibody, and RPE and NR nuclei of *Otx2^+/+^* mice were subjected to the WT assays using an Otx2 antibody. **B.** Genome distribution of Otx2 bound regions (OBRs). Upper panel: colour-coded pie chart showing peak distribution of each ChIP-seq assay compared to global genome distribution. Below, number of OBRs and peak height distribution of each assay are shown. Lower panel: percentage of peaks in defined functional domains of the genome for each assay. Colour-code is as in pie charts.

To gain insight into the respective function of Otx2 in RPE and neural retina (NR), we set out to compare the genomic targets of Otx2 in each compartment. We dissected eyes from 4–5 weeks old mice and carried out Otx2-ChIP-seq separately on RPE and NR nuclei. In order to enhance the reliability for identified Otx2-bound sites, we performed two parallel experiments using two different antibodies: first, *Otx2^Otx2−GFP/+^* knock-in mice expressing an Otx2-GFP fusion protein [13] together with an anti-GFP antibody, second, wild type mice together with an anti-Otx2 antibody (Fig. 1A). Two pairs of independent sets of data were generated, thereafter referred to as the GFP and the WT assays (Fig. 1A). A preliminary description of RPE Otx2-ChIP-seq was previously published [12]. These RPE data were subjected to in-depth analysis and comparison with neural retina ChIP-seq datasets. A total of 4-16×10^6^ sequence reads were mapped to the genome for each condition. MACS algorithm with a <1% FDR threshold was used to identify peaks, henceforth referred to as Otx2-bound regions (OBRs). NR-WT and NR-GFP assays respectively yielded 15448 and 5997 OBRs, while RPE-WT and RPE-GFP assays respectively yielded 2941 and 3766 OBRs (Fig. 1B). Control experiments with GFP antibody on wild-type mice and non-relevant Lamin-B1 antibody on wild-type mice failed to show significant sequence enrichment above background across the genome, indicating that Otx2 and GFP antibodies were both specific.

For each experiment, peak distribution across functional domains of the genome was analysed using Cis-regulatory Element Annotation System (CEAS) (Fig. 1B). For all conditions, distributions of peaks departed from the actual distribution of functional domains, indicating that peaks were not randomly located on the genome but were enriched in specific functional domains. Strikingly, both NR assays and both RPE assays exhibited their own specific characteristics. In NR-WT and NR-GFP assays, peaks were strongly enriched in promoter regions: while these regions represent about 1% of the genome, they contained 20 to 30% of the NR peaks. Enrichment of peaks was also significantly found in 5’UTR regions in NR assays. By contrast, peaks were under-represented in distal intergenic regions. RPE peaks from WT and GFP assays also showed a specific distribution pattern: peak concentrated to promoter regions, although to a lesser extent than in NR (5.3 and 7% for WT and GFP assay, respectively). RPE peaks were consistently enriched in introns and under-represented in intergenic regions. Therefore, the two independent WT and GFP assays show consistent and specific Otx2 genome occupancy landscapes in the neural retina and in the RPE, with Otx2 binding enriched in promoters and 5’UTRs in the NR and in promoters and introns in the RPE. Peak height distribution was also found to differ between NR and RPE assays: in NR assays, it showed a unimodal shape, with a moderate height. By contrast, the distribution was bimodal in both RPE assays with two distinguishable populations of low- and medium-height (Fig. 1B). The finding of NR and RPE-specific OBR signature with different global characteristics suggests that Otx2-mediated gene regulation proceeds differentially in these two retinal compartments.

### Independent ChIP-seq assays yield high confidence Otx2 bound regions datasets with characteristic features

**Figure 2:**
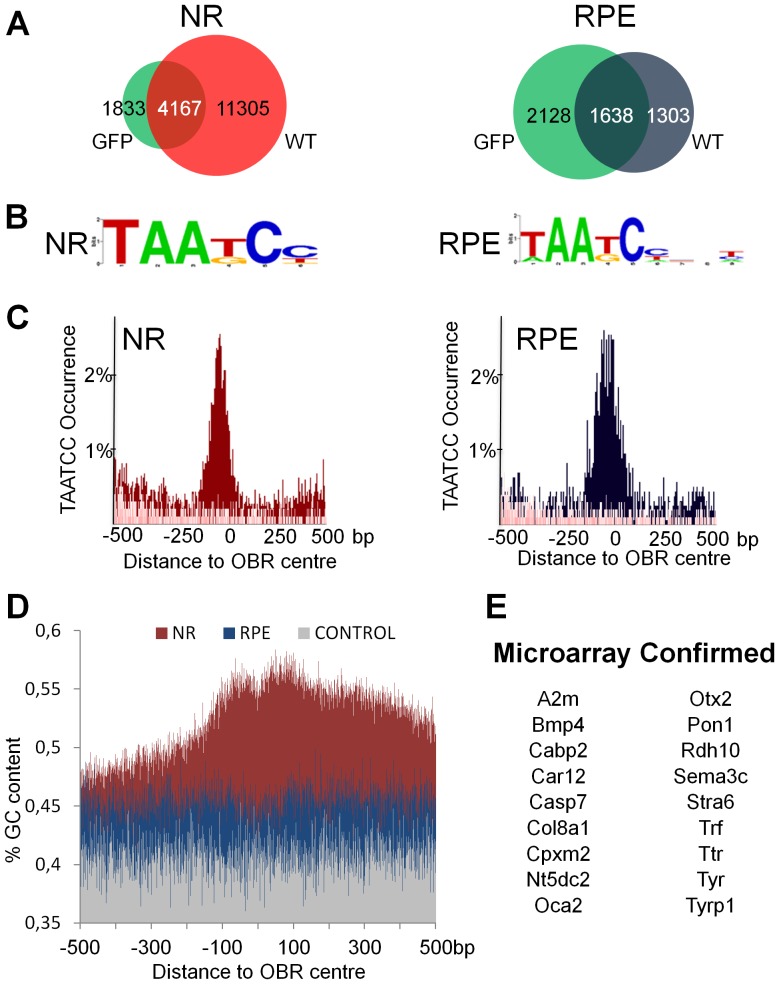
Dual ChIP-seq assays identify relevant neural retina and RPE OBR core sets. **A.** Venn diagrams showing the overlap of peaks identified in both ChIP-seq assays in each tissue. Intersections of GFP (green) and WT (red in NR, blue in RPE) assays represent two core sets of 4167 and 1638 binding sites in the NR and in the RPE, respectively. **B.** Motif enrichment analysis on the core datasets. Shown is the highest enriched TAATCC Otx2 binding consensus motif. **C.** Distribution of the TAATCC motif in 1 kb of genomic sequence around the centre of the core set of OBRs. **D.** GC content 1 kb around the centre of Otx2 bound regions in NR (red) and RPE (blue) compared to a random selection of 1000 regions in the genome (grey). **E.** RPE specific microarray confirmed genes with a called peak in their vicinity.

We next examined to which extent the double antibody approach enhanced the reliability of OBR identification, by comparing the pairs of datasets obtained in the NR and the RPE (Fig. 2A). In the NR, 69.5% of the OBRs detected in the GFP assay overlapped with OBRs detected in the WT experiment, forming a core set of 4167 peaks. We analysed the gene ontology of the corresponding 3308 closest genes using the Database for Annotation, Visualization and Integrated Discovery (DAVID) tool (Table 1). We found an enrichment for the following ontology terms: visual perception (p = 9.9×10*^−^* ^15^, Fisher Exact P-value) sensory perception of light stimulus (p = 1.6×10*^−^* ^14^), vision (p = 2.6×10*^−^* ^11^), detection of light stimulus (p = 1.1×10*^−^* ^6^), response to light stimulus (p = 8.2×10*^−^* ^6^). By contrast, such enrichment was absent in the 5903 closest genes corresponding to the non-core set of peaks. This indicates that the intersection of GFP and WT datasets in the NR corresponds to a core set of high confidence Otx2 bound regions, with strong relevance to NR function. Similarly, in the RPE, 55.6% of the OBRs detected in the WT assay were common with the OBRs detected in the GFP assay. A total core set of 1638 peaks was deduced. DAVID analysis indicated that the 1374 closest genes were enriched in specific ontology terms (Table 2): cell adhesion (p = 2.3×10*^−^* ^5^), cell junction (p = 1.0×10*^−^* ^5^), metal-ion binding (p = 3.5×10*^−^* ^4^), eye development (p = 2.2×10*^−^* ^3^), melanocyte differentiation (p = 2.4×10*^−^* ^3^) whereas these enrichments were absent or strongly reduced (p>8.8×10*^−^* ^3^) in the 2151 non-core specific closest genes. Therefore, the intersection of GFP and WT datasets in the RPE also represents a core set of high confidence OBRs with relevance to specialized function of the RPE.

**Table 1:** Ontology term enrichment in core and non-core set of the neuroretina.

Gene Ontology term	CORE	NON-CORE
visual perception	9.9E-15	absent
sensory perception of light stimulus	1.6E-14	absent
vision	2.6E-11	absent
detection of light stimulus	1.1E-6	absent
response to light stimulus	8.2E-6	absent
photoreceptor cell differentiation	2.4E-5	absent
detection of visible light	2.5E-2	absent

**Table 2:** Ontology term enrichment in core and non-core set of the RPE.

Gene Ontology term	CORE	NON-CORE
cell adhesion	2.3E-5	4.4E-2
cell junction	1.0E-5	2.9E-2
metal-ion binding	3.5E-4	absent
eye development	2.2E-3	8.8E-3
melanocyte differentiation	2.4E-3	absent
eye morphogenesis	2.0E-3	6.5E-2
pigment cell differentiation	3.2E-3	absent
melanin metabolic process	3.7E-3	absent

To characterize the structure of Otx2 bound sequences, we searched NR and RPE core datasets for enriched motifs using three different tools: Hypergeometric Optimization of Motif EnRichment (HOMER), MEME-ChIP and Motiflab (Fig. 2B and Tables S1 and S2). In the NR, all methods called the preferred Otx2 binding site TAATCC [14] with flexible T/G sequence specificity at position 4. This motif was present in 67.2% of called peaks in the NR. In the RPE core dataset, the Otx2 binding motif was present in 83.4% of called peaks, with similar flexible sequence specificity at position 4 and a 3’ extension TAATCCNNT/C. The distribution of Otx2 binding motifs in 1 kb of genomic sequence centred on all core OBRs was evaluated. In each tissue, we found a prominent peak of TAATCC sites at the centre of the OBRs (Fig. 2C), indicating that the majority of genomic fragments were captured through the binding of Otx2 protein to its preferred motif. Together, these results show that our dual ChIP-seq approach yields robust sets of OBRs with clear relevance to NR or RPE function, most of which contain the Otx2 preferred binding site at their centre.

Gene promoters and regulatory regions often lie close to CpG islands, which have a higher GC percentage than genome average. To test whether Otx2 bound regions display specific base content, we compared the percentage of GC in 1000 bases around the centre of OBRs to 1000 random genome sequences of the same length (Fig. 2D). The GC percentage of OBR-containing genomic fragments was increased in both NR and RPE, indicating that Otx2 preferentially binds to GC rich regions. In the RPE, the increase was evenly distributed around the OBRs and appeared modest (about 3%). In the NR, the GC percentage close to the OBRs was highly increased. It culminated over 55% GC in two symmetrical peaks flanking the centre of the OBRs, which might correspond to a nucleosome positioning signal [15].

To evaluate the relevance of core vs non-core datasets, we examined whether the closest corresponding genes were enriched in known Otx2 target genes. In the RPE, Otx2 target genes identified in previous microarray studies [12] were 4 times more frequent in the RPE core set than in the non-core set, with 18 Otx2 target genes in the core set, representing 78% of the RPE specific target genes identified by microarray (Fig. 2E). This analysis could not be done for NR core datasets as no Otx2 target gene specific to photoreceptor cells has been identified so far. Nonetheless, both in the NR and RPE experiments, the intersection of sequences identified in the GFP and the WT assays showed consistent global and structural traits, making them high confidence core datasets. As a consequence, further analyses were restricted to NR and RPE core datasets, which represent robust ensembles of OBRs.

### Evolutionary conservation as an indicator of relevance for Otx2-bound regions

**Figure 3:**
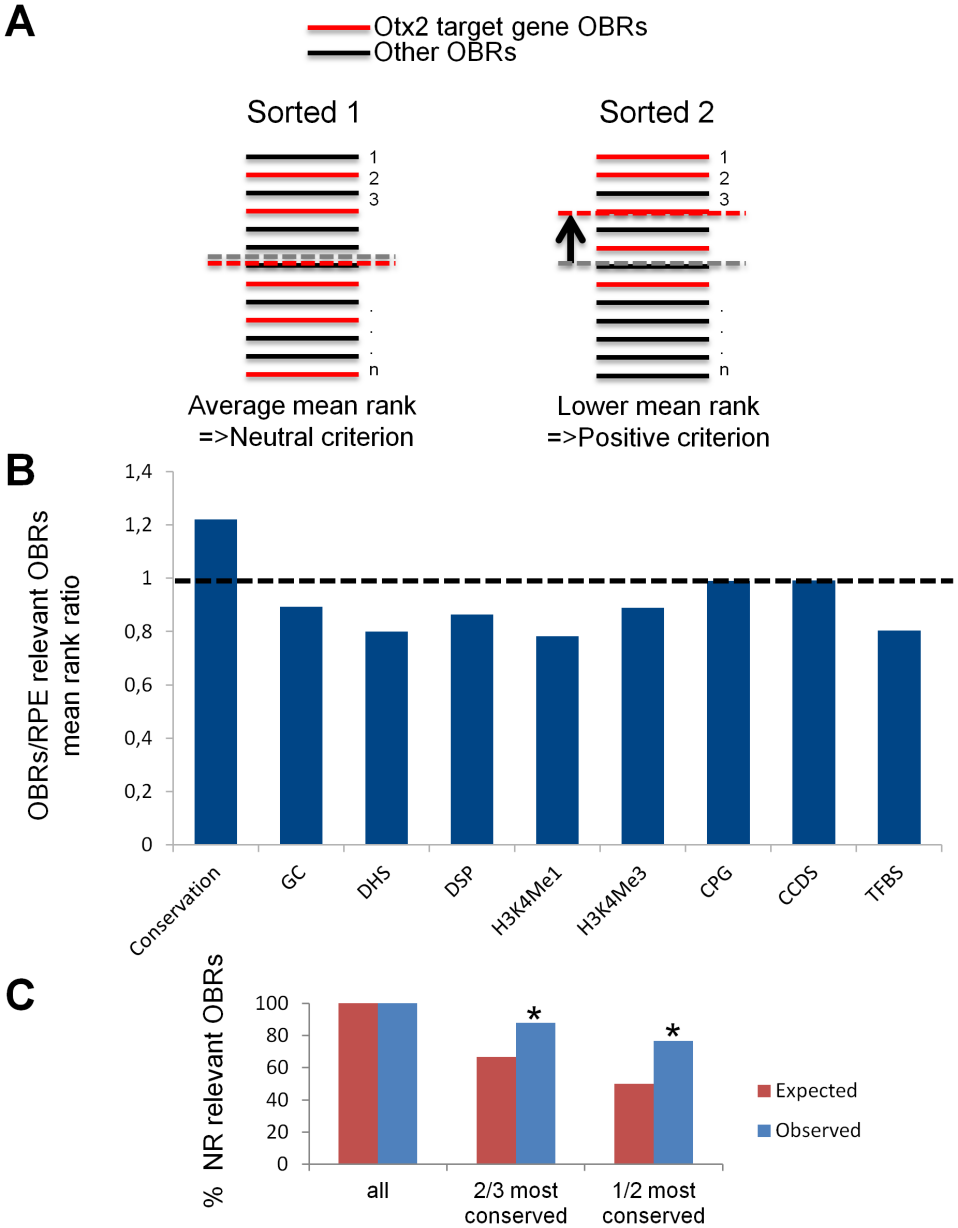
Evolutionary conservation marks OBR relevance. **A.** Principle of the relevance assay method: OBRs are sorted according to a given criterion and the mean rank of OBRs close to Otx2 target genes (red) is compared to the average mean rank of all called OBRs according to this criterion. A similar rank indicates a neutral criterion and a lower rank indicates a relevant criterion. **B.** Shown is the ratio of all OBRs mean rank to the mean rank of OBRs close to microarray confirmed Otx2 target genes according to evolutionary conservation, GC content, DNase hypersensibility (DHS) DNase sensibility and sensibility peaks (DSP), histone H3 lysine 4-mono- and tri-methylation (H3K4Me1, H3K4Me3), CpG islands (CPG), Consensus Coding sequences (CCDS) and known transcription factor bound regions (TFBS). The dashed line at the value of 1 represents the neutrality of all criteria. **C.** Application of the conservation criterion to the NR: expected and observed percentage of OBRs close to genes relevant to neural retina Gene Ontology terms among the 2/3 and 1/2 most conserved OBRs.

Among the thousands of genome sites bound by transcription factors, no clear indication has emerged so far that could help recognizing those that have prominent regulatory role. We used our knowledge of Otx2 direct target genes to evaluate whether additional features such as chromatin marks or sequence properties could help predicting the relevance of OBRs. The rationale was the following: if a given criteria is relevant to cis-active function of an OBR, then target-gene OBRs should have a lower rank according to this criteria (Fig. 3A). We used the list of previously identified Otx2 direct target genes to sample criteria. As most of these genes were RPE-specific, we restricted our analysis to the RPE OBR core dataset. Figure 3B shows that only sequence conservation led to a mean rank ratio above 1, indicating a lower mean rank of the selected genes. For all other criteria, the relative mean rank of OBRs close to Otx2 target genes remained equal to or below 1. Thus, sequence conservation is an indicator for OBR relevance in this experiment, and might help distinguishing functional binding sites in ChIP-seq experiments. To assess the predictive value of this criterion, we applied it to the NR core dataset. We tested whether OBRs close to genes with relevant ontology terms were enriched among the most conserved OBRs (Fig. 3C). When considering the 2/3 most conserved OBRs in the NR, we observed that 90% of OBRs close to relevant NR genes were present, a proportion significantly superior to the expected one. When considering the 50% most conserved OBRs, 75% of OBRs close to relevant NR genes were included, an even more remote proportion from the expected one. Khi^2^ tests supported the statistical significance of these results. Hence, filtering the most conserved OBRs also distinguishes relevant genes in the NR.

### Otx2-bound regions control different genetic repertoires in NR and RPE

**Figure 4:**
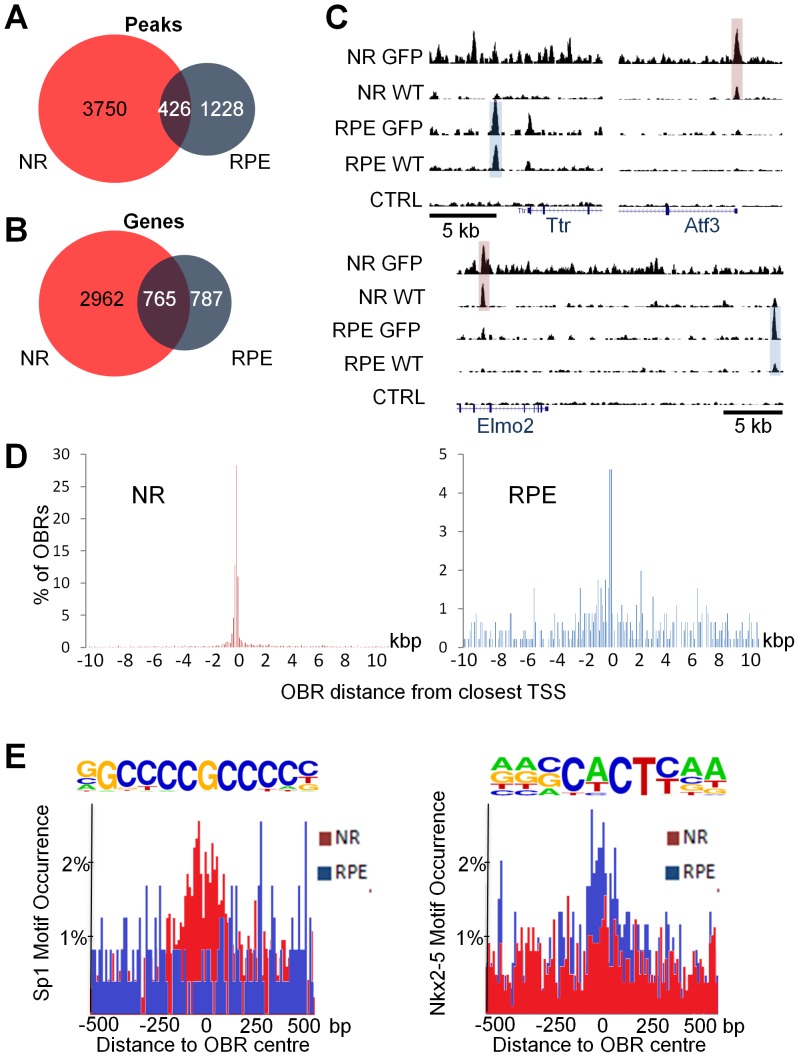
Contrasted Otx2 genome occupancy in neuroretina and RPE. **A.** Venn diagram showing the overlap of OBR core sets in NR (red) and RPE (blue). **B.** Venn diagram showing the overlap of the closest genes to core set OBRs. **C.** Representative examples of OBR localization in RPE-specific (Ttr), NR-specific (Atf3) and common (Elmo2) genes. Shown are browser captures for the 4 ChIP-seq assays and control. The gene is indicated in blue at the bottom. **D.** Distribution of distance from the closest transcription start site (TSS) in NR and RPE for all OBRs lying within 10 kb from a known TSS. **E.** Distribution of Sp1 and Nkx2-5 motif occurrence 1 kb around the centre of OBRs in the NR (red) and the RPE (blue).

Although Otx2 is expressed in photoreceptor and bipolar cells of the adult mouse retina, its role in the NR, especially in photoreceptors, remains unclear. Previous studies have shown that RPE-specific rescue of Otx2 expression following whole knockout is sufficient to maintain healthy photoreceptors, suggesting that Otx2 is dispensable in these cells. This raises three hypotheses: i) either Otx2 transcription factor has no function in the NR, or ii) these functions are not essential and do not lead to any visible phenotype, or iii) compensatory mechanisms in the NR mask the effects of Otx2 deletion. All these hypotheses imply that Otx2 would have different functions in NR and RPE. To test this idea, the two confident NR and RPE core OBR sets were compared to determine whether Otx2 binds to the same locations in both tissues (Fig. 4A). Out of their respective 4167 and 1638 OBRs, NR and RPE core datasets shared only 426, indicating that Otx2 occupancy is largely distinct it the two tissues. Surprisingly, when looking at the closest gene to each bound region, and comparing genes close to OBRs instead of binding regions in NR and RPE, the overlap was notably increased. From the 426 overlapping peaks, corresponding to 381 genes, common closest genes to Otx2 bound regions were brought up to 765 (Fig. 4B). An additional group of 384 genes with an OBR in their vicinity in both NR and RPE was hereby identified, with their OBR located at different positions in each tissue. An example for each case is shown in Figure 4C. Thus, Otx2 binds to the proximity of mostly different genes in the NR and in the RPE, and for half of the few genes that share an OBR in both tissues, the site of binding occupies a different position. This is in favour of Otx2 regulating very different gene repertoires in different retinal layers.

The observation of a different OBR location for 384 genes in the NR and in the RPE suggested potential difference of Otx2-bound cis-regulatory elements organization in both tissues. For instance, several PR specific genes have been shown to bear important cell-specific regulatory elements very close to their transcription start site (TSS) [16]–[19]. To test whether this observation extended to all genes with an OBR, we made a systematic evaluation of the position of OBRs relative to the closest TSS for all OBRs located from –10 kb to +10 kb of a known TSS (Fig. 4D). This revealed a major difference between NR and RPE. In the RPE, OBRs were widely distributed around the TSS, with a modest but clear increase in most proximal (<200bp) region around the TSS. Around 10% of OBRs located in this region, the other 90% being localized homogeneously along those 20 kb. In the NR, OBRs preferentially localized in the proximal region, with over 40% tightly associated to the TSS. This showed that Otx2 binds to regulatory motifs with flexible distance to TSS in the RPE. In contrast, in the NR, Otx2-bound regulatory motifs have a much more prevalent proximity to TSS, which may be dictated by the short size of photoreceptor specific promoters.

Along with different chromatin structure and promoter organization, differential protein associations could contribute to select specific Otx2 binding sites, explaining the differences we observed in both tissues. To explore this possibility, we used HOMER motif enrichment analysis to search whether motifs recognized by other transcription factors could be associated to the OBRs (Fig. 4E). In the NR, an associated Sp1 site was found in 19.6% of OBRs. As Sp1 sites are GC rich, we thought their enrichment might be artificially increased due to random appearance in CpG islands where Otx2 is bound. However, we found Sp1 sites were still enriched in OBRs not located in CpG islands (p = 1×10*^−^* ^32^), supporting the significance of this result. Other enhancer sites for GFY and NFY were enriched to a lesser degree (Table S1). In the RPE, an enrichment of Nkx2-5 (29.7% of OBRs), Lhx3 (24.5% of OBRs) and Pax7 (3.0% of OBRs) transcription factor binding site was found in Otx2 bound regions (Table S2). To further characterize the relationship between the enriched motifs and the Otx2 binding motif, we examined the distribution of Sp1 and Nkx2-5 motifs 1 kb around the centre of the OBRs. Sp1 motif clustered to the centre of OBRs only in NR while Nkx2-5 motif peaked around the centre of RPE OBRs only. Conversely, the distribution of Sp1 and Nkx2-5 motifs was totally random in RPE and NR OBRs respectively (Fig 4E). Since the Otx2 binding TAATCC motif occupies the centre of OBRs in both retinal tissues (Fig. 2C), this demonstrates a close and specific association of Otx2 and Sp1 binding sites on NR cis-regulatory elements and of Otx2 and Nkx2-5 binding sites on RPE cis-regulatory elements. The data are consistent with the possibility that specific transcription factor-Otx2 combinations select different regulatory motifs in the NR and in the RPE.

In conclusion, the strong difference of Otx2-bound regulatory element architecture in RPE and NR and the difference of gene sets (Table S3) with an OBR in each of these retinal tissues as well as the presence of specific enriched motifs for other transcription factors clearly supports a different role for Otx2 in the NR and in the RPE.

### Otx2 and Crx may act redundantly in the neural retina

Otx2 does not bind to the same gene regulatory regions in the NR and the RPE, and therefore might have a different, yet unknown function in the NR. The rescue of photoreceptor degeneration in Otx2 knockout retinas by the RPE-restricted expression of Otx2 shows that there is no crucial need for Otx2 in the NR. One reason for this apparent lack of function could be that Crx, an Otx2 paralogue expressed in the same cell types of the NR as Otx2 but not in the RPE, could compensate for the lack of Otx2. To explore this possibility, we used previously published Crx Chip-seq data [17] to compare Otx2 and Crx genome occupancy. In order to homogenize all datasets, the Crx data was re-processed with the same analysis pipeline as the Otx2 data using MACS2 to call peaks.

**Figure 5:**
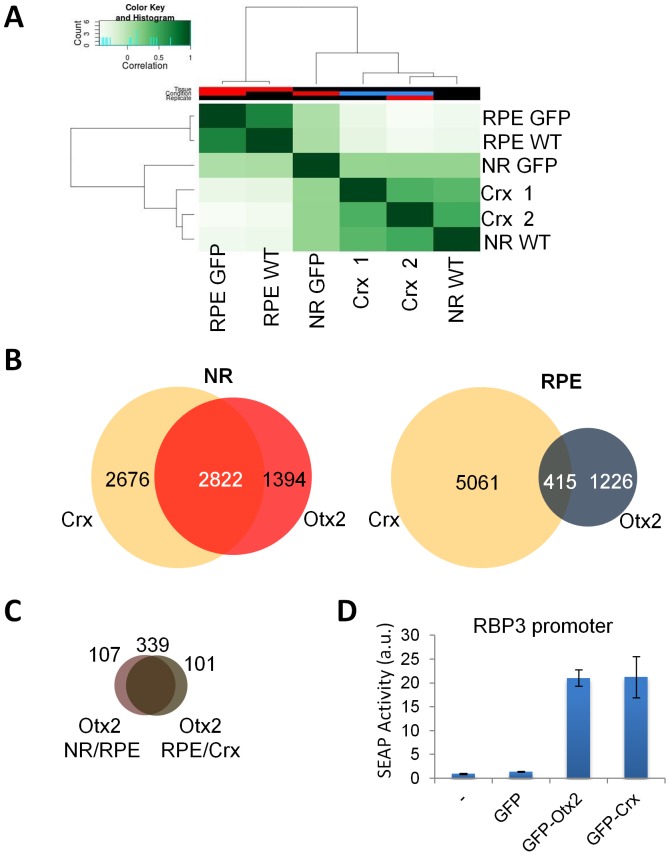
Otx2 and Crx redundancy in the neuroretina. **A.** Heatmap and dendrogram representation of Diffbind clustering of the indicated ChIP-seq experiments. **B.** Venn diagrams showing overlapping Crx bound regions (CBRs) and OBRs in the NR and in the RPE. CBRs represented the intersection of both Crx ChIP-seq replicates. **C.** Venn diagram showing the overlap between the above 415 common OBRs/CBRs in the RPE (grey) and the 426 OBRs common to RPE and NR (purple) shown in Fig. 4A. **D.** Otx2 and Crx transactivation of the *RBP3* promoter.

First, a global comparative analysis of Otx2 and Crx raw ChIP-seq data was carried out using the bioconductor DIffBind tool, clustering multiple ChIP-Seq experiments together into a correlation heatmap (Fig. 5A). Both Otx2 RPE WT and GFP ChIP-seq data clustered together in a very close group, and the two Crx replicates also clustered together in another close group. Interestingly, in this analysis, the Otx2 NR WT and GFP ChIP-seq data bracketed the Crx group, showing closer similarity to this Crx group than to the Otx2 RPE group. This indicated that Otx2 genome occupancy in the NR was more similar to Crx genome occupancy that to Otx2 genome occupancy in the RPE.

Next, the Otx2 NR and RPE core datasets were compared to the Crx ChIP-seq data in detail (Fig. 5B). Peak location analysis revealed a very large overlap between Otx2 NR and Crx peaks with 2822 overlapping peaks. On the contrary, Otx2 RPE and Crx only shared 415 peaks, 339 of which already belonged to the intersection of NR and RPE peaks (as shown in Fig. 4A) and were thus not RPE specific ones (Fig. 5C). Finally, in the RPE, only 101 genome sites representing 6% of OBRs were common with Crx bound regions whereas in the NR, 67,7% of Otx2 bound regions were also bound by Crx. This supports the view of a redundant activity of Crx and Otx2 in the NR that may explain the absence of phenotype in NR-specific knockout of Otx2.

To test whether Otx2 and Crx may display similar regulatory activities and hence compensate each other, we compared their ability to stimulate transcription in a transient transfection assay (Fig. 5D) A reporter construct containing the *Rbp3* promoter, an Otx2 target [20] fused to the secreted alkaline phosphatase (SEAP) reporter gene was transfected into HeLa cells along with expression vectors for GFP, GFP-Crx or GFP-Otx2 fusion proteins. Both Otx2 and Crx proteins stimulated *Rbp3* promoter transcription to similar levels, causing a 20-fold increase of phosphatase activity compared to controls. This demonstrates that Otx2 and Crx can have identical activity on target promoters.

Together, these results show that, in the neural retina, both Otx2 and Crx transcription factors can bind to many identical places on the genome and have similar activity *in vitro*, making it is very likely that they exert redundant activities in the NR. On the contrary, in the RPE, where Otx2 binds to essentially different sites, no redundant Crx activity is present. Therefore, while Otx2 ablation in the RPE causes dramatic phenotypical changes, it is expected to have much less impact in the NR because of Crx redundancy.

## Discussion

Although it has been long recognized that regulatory proteins may control different activities in different contexts, little is known about how this is achieved. This study provides the first comparative genome-scale analysis of the binding of a transcription factor in two different tissues. By performing dual ChIP-seq experiments on Otx2 in two contiguous but physiologically different retinal tissues, the neural retina and the retinal pigment epithelium, we present strong evidence that this transcription factor regulates mostly non-overlapping gene sets in both retinal compartments.

Otx2 mainly acts as a transcriptional activator. This is supported by the fact that early deregulated genes after Otx2 knockout are almost all down-regulated [12], and by previous studies [21], [22]. Therefore, to perform distinct functions in both NR and RPE, Otx2 must activate different target genes. The dual ChIP-seq experiment using two different antibodies is a unique approach, which guarantees robust OBR identification. Identified binding sites are consistent and their intersection is a reliable dataset. Thanks to this strategy, we show that in the adult eye, Otx2 has extremely different properties in two distinct cellular layers where it is expressed: the RPE and the NR. OBR-bearing genes have relevant tissue-specific ontologies for both tissues. In the NR, Otx2 binds to cis-regulatory elements with high GC content that lie very close to the TSS. In the RPE, Otx2-bound cis-regulatory elements have a broader distribution, relative to the TSS. This suggests that Otx2 exerts different activities in NR and RPE by regulating differential genetic networks. Accordingly, OBRs are very different in both tissues, with poor overlap.

Two elements may contribute to the binding diversity observed in mouse eye. First, chromatin structure may influence gene accessibility [23]. Rod photoreceptors have very specific chromatin architecture [24], [25] with their heterochromatin condensed at the centre of the nuclei, and a peripheral ring of euchromatin around. Secondly, different protein associations can target a given transcription factor to different genomic sites. Interestingly, Otx2 binds in the vicinity of Sp1 binding sites in the NR, whereas it binds close to other transcription factors binding sites in the RPE. Although we identified binding sites for Lhx3 and Nkx2.5 in the RPE, the cognate transcription factors remain to be determined, as other RPE-expressed transcription factors might share similar binding motifs. The ubiquitously expressed Sp transcription factors are known to regulate photoreceptor specific genes through synergistic interaction with cell-specific regulatory proteins. For instance, the Otx-related Crx protein interacts with Sp1, Sp3 and Sp4 in photoreceptors to achieve elevated expression of phosophodiesterase-beta and rod opsin genes [26]. Our findings suggest that Otx2 may also synergise with Sp proteins to regulate photoreceptor-specific genes.

One unexpected observation is that, in NR and RPE, Otx2 binds to different regions even in the vicinity of the same genes. Both tissues derive from the same neuro-epithelium and their fate can be changed at early stages. In *Otx1^−/−^; Otx2^+/−^* embryos, upon low dosage of Otx proteins, RPE cells adopt an NR identity [6], [27]. On the contrary, Otx2 overexpression in the NR activates an RPE-specific gene network [28]. At adult stage, both tissues express Otx2, but use it in different means. In the RPE, Otx2 regulates genes involved in melanogenesis, visual cycle, pH regulation, and metal concentration homeostasis. In the mature NR, Otx2 has no known functions, but it exhibits much different genome occupancy. This may reflect tissue specific specialization with cell equipment and chromatin organization creating gene regulatory conditions that profoundly differ, even for the same genes. Such a regulatory rewiring might explain the loss of tissue plasticity in species with reduced regenerative capacity [29]. Although human pluripotent stem cells can be cultured to induce photoreceptor differentiation [30], and rat ciliary-derived cells can be transformed into cells showing a photoreceptor phenotype [31], the cellular plasticity of mammalian retinal cells is by far not as potent as in Zebrafish [32] or Goldfish [33]. It would be of high interest to test whether OBR localisation in RPE and NR of organisms with high retinal regenerative capacities is more similar than in mice, as an indication of greater plasticity of cell types.

Transcription factors ChIP-seq experiments generally reveal thousands of binding sites. Are all of them truly active? That a given transcription factor could regulate the expression of thousands of genes cannot easily be reconciled with the fine-tuned gene expression observed in complex organisms. It is therefore important to find out diagnostic elements that can help predict functional binding sites. One study has explored whether specific features of the primary sequences bound by Crx have informative value [34]. Comparison of heterologous constructs containing Crx Bound Regions (CBRs), to unbound regions containing Crx motif in a large scale *ex vivo* expression assay indicates that a high GC content of DNA regions bound by Crx is a cue of their functional activity. We tried a different approach. Starting from our knowledge of direct Otx2 targets in the RPE [12], we examined how the corresponding OBR ranked among all OBRs sorted according to several criteria. This analysis did not pick-out GC content as a relevance criteria. This may be due to the fact that we did the analysis with RPE OBRs, as our target gene list was restricted to this cell type. The situation may be different in the NR, notably in photoreceptor where short promoters may concentrate Otx2 or Crx binding sites together with GC-rich Sp binding sites. However, we found that evolutionary conservation positively correlates with functionally active OBRs, which makes sense, as regulatory sequences are known to be under selection constraints [35].

Another way to identify which transcription binding sites, among the thousands revealed by ChIP-seq, are functionally important would be to identify those that are engaged in enhancers or transcription hotspots. As chromatin marks and protein complexes present in these structures are better and better understood, they will provide new means to annotate transcription factors ChIP-seq datasets.

Otx1, Otx2 and Crx have a common evolutionary origin. In *Drosophila*, a single ortholog, Orthodenticle (otd) performs all Otx functions in eye and retina development. Mammalian Otx proteins can rescue some defects of *otd* mutant flies although to various extents [36]. These differences reflect the fact that mammalian *Otx* genes have acquired specialized sub-functions through evolution. Indeed, each member of the family has specialized activities, some of which being unique. For instance, Otx1 can rescue gastrulation defects in *Otx2* knockout mice but fails to develop anterior head [37]. Symmetrically, Otx2 rescues epilepsy and corticogenesis abnormalities in *Otx1* knockout mice but fails to recover the lateral semicircular canal of the inner ear [38]. Whether Crx is able to replace Otx1 or Otx2 activities has not been tested so far. In the adult mouse, Otx2 and Crx are both expressed in bipolar cells and photoreceptors. Our findings that both transcription factors share *in vivo* binding and *in vitro* transactivation properties argue in favour of their redundancy in the neural retina and hence bring a simple explanation for the absence of photoreceptor specific *Otx2* knockout phenotype.

## Methods

### Ethics Statements

All experiments were conducted under UE guidelines approved by local and state ethical committees. TL received the authorization to experiment #06-261 from the DDPP of the Préfecture des Alpes Martimes, France, and protocols used in this study were approved by the local ethical committee CIEPAL-Azur (permit #Nce/2011-25).

### Chromatin Immunoprecipitation

For RPE ChIP, 40 mouse eyes were dissected to remove cornea, lens and neural retina. RPE/choroid eye cups were directly cross-linked in 1% formaldehyde in DMEM at room temperature for 10 min then quenched by adding glycine at a final concentration 125 mM and incubated at room temperature for 5 min. Eye cups were washed twice in cell wash buffer (20 mM HEPES pH 7.4; 150 mM NaCl; 125 mM Glycine, 1 mM PMSF). RPE/choroid nuclei (around 5 million) were then isolated with a dounce (pestle B) in cell lysis buffer (20 mM HEPES pH 7,4; 1 mM EDTA; 150 mM NaCl; 1% SDS; 125 mM Glycine; 1 mM PMSF). Eyecups were removed and RPE/choroid nuclei suspension was obtained. The rest of ChIP procedures were performed as described on Farnham Lab web site (http://farnham.genomecenter.ucdavis.edu/pdf/FarnhamLabChIP%20Protocol.pdf).

For neural retina, the eyes of 6 mice were dissected in ice-cold PBS. Cornea and lens were removed. RPE and neural retina were mechanically separated at the level of photoreceptor outer segment, avoiding nuclei cross-contamination between both tissues. Neural retinae were processed following the same protocol than for RPE ChIP. On average, around 100 million cell nuclei were obtained.

An antibody raised against OTX2 (R&D systems, Minneapolis, MN, USA) and an antibody raised against GFP (Abcam, Cambridge, UK) were used to precipitate chromatin–Otx2 complexes from wild type mice in the WT assay, and chromatin-Otx2-GFP complexes from Otx2^Otx2*−*GFP/+^ mice in the GFP assay, respectively. An antibody raised against Laminin B (Santa-Cruz, California) and the antibody raised against GFP were applied to wild type chromatin and used as controls for WT and Otx2-GFP assays, respectively. The final DNA precipitates were dissolved in 20µl of TE (20 mM Tris–HCl pH 8.0, 1 mM EDTA).

### ChIP-seq, clustering of sequence reads and identification of OBRs

ChIP-seq experiments were performed according to standard protocols as previously described [39]. All four samples of ChIP dual-assay were processed for ChIP-seq by IGBMC sequencing platform (IGBMC, France). Quantity and quality of DNA was ensured before processing by Qubit dsDNA HS Kit (Invitrogen) and bioanalyzer 2100 (Agilent). For each sample, 10 ng of DNA was used to generate ChIP-DNA library. Libraries were analyzed by bio-analyzer 2100 (Agilent), then, were massively sequenced by Illumina GAIIx sequencer, generating 40 million reads of 35bp length per sample, on average. Raw reads data were analyzed and aligned on mouse genome mm9 by CASAVA v1.8. All data were deposited on GEO, under accession number GSE54084.

Clustering for WT and Otx2-GFP assays was performed by first generating density (.wig format files) counting the number of tags in a 25pb sliding window for each ChIP-seq data set. Peak detection was performed using the MACS software (http://liulab.dfci.harvard.edu/MACS/) [40] in version 2.0 at a q-value of 0.01, which reproduced most of the MACS 1.4 called peaks in each experiment with 3 times higher precision. Overlapping OBRs were identified and annotated using PeakAnalyzer v1.4 (http://www.ebi.ac.uk/bertone/software.html), with respect to the coordinates of the beginning and end of RefSeq transcripts. Due to possible overlap of one peak from a dataset with two peaks from another, total peak number may slightly vary.

### Comparative ChIP-seq Analysis

For each ChIP-seq dataset, Cis-regulatory Element Annotation System 1.0.2 [41] was used to map called peaks on genomic regions. Further comparative analysis were yielded using R [42]. Proportional Venn diagrams were plotted using made4 [43] and VennDiagram [44] packages. ChIP-seq clustering was performed using Diffbind tool [45].

### Peak Ranking

Motiflab tool [46] was used to load UCSC tracks and evaluate region coverage of each criterion. Peaks were then sorted according to these criteria. Global mean rank varied according to the number of peaks having the same criterion value. The selected gene’s mean rank was compared to this global mean rank. A ratio above 1 indicates a good confidence criterion. Conservation score was gathered from the multiz30way UCSC track, consisting of a multiple alignments of 30 vertebrate species from mouse to zebrafish. Sequences were sorted according to the mean Phastcons score [47] of the OBR. The DNaseHS peaks, H3K4me1 and H3K4me3 scores were based on peak regions from many different cell types, and the TFBS ChIP-seq score was based on data for many different transcription factors. OBRs were sorted using the Motiflab tool according to 10 criteria: GC percentage, conservation, number of TAATCC motifs, CCDS (Consensus CoDing Sequences), H3K4 mono- or tri-methylation, DNAse hotspots, TFBS, CpG islands. In each sorted list, the mean rank of all OBRs relative to the mean rank of OBRs close to Otx2 regulated genes was calculated. This ratio is expected to be higher than 1 for any relevant criteria.

### ChIP-seq motif Analysis

Three independent motif enrichment methods were used on the neural retina and RPE specific overlapping “core” set : MEME-ChIP software Version 4.9.0 [48], Homer 4.3 [49] and Motiflab [46]. The latter tool was also used for OBR ranking and GC content analysis. For Sp1 motif, correlation with CpG was studied by splitting the dataset in three subsets: i) close to the promoter (1 kb) with CpG island; ii) close to the promoter (1 kb) without CpG island; iii) distal OBR (>1 kb).

### Cell Culture

HeLa cells were cultured at 37°C, 5% CO2 and 95% humidity in Dulbecco Modified Eagle's Medium (Invitrogen, Carlsbad, CA, USA) supplemented with 10% fetal calf serum (Perbio, Helsingborg, Sweden), 50 units/ml penicillin, 50 µg/ml streptomycin and 2 mM L-glutamine (Invitrogen).

### Reporter gene assay

Transcriptional activity of the Otx2 and Crx proteins was assayed using the region –66 to +68 of the *IRBP* promoter cloned into the pSEAP2-basic vector (BD Biosciences, Palo Alto, CA, USA). The pSEAP2-Basic (promoter-less) and pSEAP2-Control (SV40 promoter) vectors were used as negative and positive controls respectively. In standard assays, 10^5^ HeLa cells per well were seeded in 24 wells plates and transfected by the CaPO_4_ method with unless indicated, 1 µg IRBP-SEAP, 0.25 µg GFP or GFP-Otx2 or GFP-Crx expression vector and 0.1 µg beta-galactosidase expression vector. After 40 hours incubation, secreted alkaline phosphatase (SEAP) and beta-galactosidase activities were measured. SEAP activity was normalized with beta-galactosidase activity. Normalized SEAP background in the absence of Otx2 expression vector was taken as 1 fold activation. Three independent experiments were performed in duplicate to generate each data.

## Supporting Information

Table S1 HOMER enriched motifs in the NR.
(XLSX)

Table S2 HOMER enriched motifs in the RPE.
(XLSX)

Table S3 Closest genes lists.
(XLSX)
